# Is nocturnal enuresis a predisposing factor for the overactive bladder?

**DOI:** 10.3906/sag-1604-116

**Published:** 2019-06-18

**Authors:** Nicolaie SUDITU, Irina NEGRU, Adelina MIRON, Bogdan NOVAC, Catalin CIUTA

**Affiliations:** 1 Department of Surgery, Arcadia Hospital, Iaşi Romania; 2 Department of Urology and Kidney Transplant, “Dr. C.I. Parhon” Clinical Hospital, Iaşi Romania

**Keywords:** Nocturnal enuresis, overactive bladder, predisposing cause, risk factors

## Abstract

**Background/aim:**

This study aimed to perform a limited observational study to ascertain whether there is statistical support that nocturnal enuresis (NE) is a predisposing factor in the development of overactive bladder (OAB).

**Materials and methods:**

The authors recruited patients diagnosed with OAB over a period of twelve months, and those who declared a history of NE were asked additional questions regarding the features of their NE.

**Results:**

A total of 285 patients were diagnosed with overactive bladder, and 98 (34.38%) of them had previously displayed NE symptoms that had diminished before reaching the median age of 9.83. Separation of patients by sex revealed a male majority (58.16%). Additionally, most patients had urban origins (75.51%). The median time span from remission of NE to diagnosis of OAB was 24.79 years, and the median age at which patients began to suffer was 31.80 years. Behavioral factors (smoking, alcohol consumption) and psychological and infectious factors (past history of urinary tract infection) were identified at varying degrees.

**Conclusion:**

The presence of NE in a third of the patients who developed over time OAB and the earlier onset of OAB for these patients suggests a causal physiopathological relationship between NE and OAB. The preponderance of urban patients confirms the existence of acquired urban triggering factors of OAB (nutritious, social, or professional).

## 1. Introduction

Nocturnal enuresis (NE) is potentially a genetically transmitted pathology, and it occurs relatively frequently; 5%–10% of children 5 to 7 years old, with a preponderance of male children, exhibit NE symptoms [1]. Out of all NE cases, 15% will achieve bladder control each year; thus, only 1% of NE patients continue to exhibit symptoms at the age of 18 years old [2]. 

The etiology of NE has not been fully described and a cause can only be identified in a small percentage of patients [3]. The locus coeruleus is located at the level of the mesencephalon, being the noradrenergic center responsible for waking from sleep. It overlaps with the micturition center, which is located at the level of the pons of the brainstem, and also has neural connections with the hypothalamic cells that produce antidiuretic hormone [4–6]. It would seem that the determinant morphopathological element of NE is a series of anomalies in these areas of the brainstem that essentially delay their functional maturation [3,7]. These anatomical alterations would be responsible for provoking the following three causal mechanisms from a physiopathological point of view: nocturnal polyuria due to nocturnal deficit of antidiuretic hormone, nocturnal overactive bladder (OAB), and a persistently high level of nervous stimulation of the aforementioned centers.

OAB is a relatively frequently manifested pathology, with a prevalence between 12.8% and 16.9% in women and 10.8% and 16% in men [8,9]. It results in a considerable decrease in the patients’ quality of life, even though it is not life-threatening [10]. 

OAB’s etiopathogeny is not fully understood, but three theories of the triggering mechanisms of OAB have been proposed: the neurological theory, the myogenic theory, and the theory of the alteration of peripheral autonomous activity. The neurological theory suggests that detrusor muscle hyperactivity is either due to decreased suprapontine inhibition (because of damage to the axonal pathways of the spinal cord), or to altered sacral activity at the synaptic level (along with the creation of primitive spinal bladder reflexes through the stimulation of the associated C-fiber bladder afferent neurons) [11]. The myogenic theory maintains that altered smooth muscle cells of the detrusor, which lack normal innervation, have altered membrane potential, which causes spontaneous contractions and facilitates the propagation of the contraction of the other normal muscle cells [12]. The theory of the alteration of the peripheral autonomous activity claims that certain morphofunctional modules exist at the level of the detrusor, being under the control of the plexus located in the intramural ganglia and interstitial cells, and dysfunction in these modules causes localized hyperactivity, along with the alteration of the inhibition–stimulation equilibrium at the level of the detrusor [13]. 

The existence of so many theories of the etiopathogeny of NE and of OAB indicates the lack of knowledge regarding the mechanisms of these conditions, without denying the possibility that etiopathogenic associations/superpositions exist. However, we believe that all of these theories are essentially based on a neurological etiopathogenic mechanism, regardless of whether we support the NE mechanism theories of nocturnal polyuria or altered excitability at the pons level, or the neurologic or myogenic theories of the mechanism of OAB. Thus, the appearance of OAB in adulthood in people who exhibited NE during childhood would make sense, especially in cases with favorable triggering factors of OAB [7,14]. A literature review revealed only a few studies suggesting a possible temporal succession between NE during childhood and various bladder dysfunctions suffered in adulthood. Some authors found an association between NE and urinary incontinence or other bladder dysfunctions in adult life [15,16]. Other authors highlighted the NE–nocturia succession and Yeniel was the only one, to our knowledge, who described NE as a risk factor for developing OAB in adulthood [17,18]. 

The aim of the present study was to evaluate whether there is a significant relationship between NE and OAB and what the risk factors might be for the occurrence of OAB in adulthood. 

##  2. Materials and methods.

### 2.1 Definition of NE and OAB 

NE is defined by the International Children’s Continence Society as urinary incontinence experienced during sleep by children over 5 years of age [19]. OAB is defined by the International Continence Society as a bladder pathology that features mictional urgency and frequency and nocturia that are or are not associated with urinary incontinence, in the absence of UTI or other obvious pathology [20].

### 2.2. Study design

The study period was 12 months, from January to December 2014. All patients with OAB included in the study were diagnosed only by the authors in their offices (public and/or private) using the same criteria and protocol. All the patients were over 18 years old and gave permission for their medical data to be used in this study.

The inclusion criteria for this study were patients diagnosed with OAB who confirmed NE in their medical history. 

The OAB patients’ diagnosis involved a standard protocol consisting of a careful medical history, symptom questionnaires (OAB Questionnaire: OAB-q), physical exam, urinalysis and/or urine culture, and renal and bladder ultrasound including postvoiding residual assessment [21]. In some patients, additional procedures (cystoscopy, urodynamics) were necessary to validate an OAB diagnosis. When the patients’ medical histories were recorded, the authors specified whether the patients had previously experienced NE, and when this was the case, the relevant clinical features were also recorded, including frequency, age of remission, behavioral factors, associated pathology, length of asymptomatic period, and period of time between manifestation of symptomology and receiving the OAB diagnosis. 

For the patients who declared NE, the diagnosis was obtained by history-taking, including in the study those patients who declared persistence of NE beyond the age of 6 years. 

### 2.3. Statistical analysis 

We calculated the confidence intervals with the standard confidence level of 95% for proportions. This constitutes the numeric interval that encompasses the real proportion of the studied population, taking into account sampling error. To eliminate errors caused by the small samples, size, and ratios nearing extremes, i.e. 0 and 1, we used the Clopper–Pearson method. We performed the calculations using application R, version 3.1.2.2015, module PropCis, function exactci, and SPSS 17 (analysis and nonparametric tests).

## 3. Results

During the study period, 285 patients were diagnosed with OAB by the authors. Of all these patients, 98 (34.38%) had experienced NE during childhood/adolescence/adulthood, and these patients formed the study group (95% CI: 0.2888400–0.4021652). There was a male majority in our study cohort, with a total of 57 males (58.16%) (95% CI: 0.4776590–0.6805376) compared with 41 female patients (41.83%) (95% CI: 0.3194624–0.5223410). Separation based on environment of origin showed that the majority of patients were from urban areas, with 74 such cases (75.51%) (95% CI: 0.6578697–0.8363566). Only 24 patients (24.49%) were from rural settings (95% CI: 0.1636434–0.3421303). 

As shown in Table 1, in all of these cases, NE diminished in the age range of 6 to 30 years, with a median value and standard deviation of 9.83 ± 3.08 years, whereas diagnosis of OAB was observed in an age range of 14 to 32 years from the time of NE remission, with a median interval of 24.79 years (standard deviation: 4.18 years). Classification based on the age at OAB diagnosis (Table 1) was performed for objective observation of groups of patients in their 20s, 30s, and 40s. The majority of patients, 61 (62.24%) (95% CI: 0.5188474–0.7184395), were diagnosed in their 30s, with a median age of 34.51 years (standard deviation: 5.81 years). The interval between the onset of bladder dysfunction and OAB diagnosis (Table 1) ranged from 6 months to 20 years, with a median value and standard deviation of 2.71 ± 2.713 years. 

**Table 1:** Patient data.

Years	Patients number by NE remission age (%)	Patients number by interval of NE remission- OAB diagnosis (%)	Patients number by age at OAB diagnosis (%)	Patients number by interval of OAB clinical onset-OAB diagnosis (%)
1				41 (41.83%)
2				24 (24.48%)
3				10 (10.2%)
4				7 (7.14%)
5				5 (5.1%)
6	9 (9.18%)			5 (5.1%)
7	9 (9.18%)			
8	12 (12.24%)			3 (3.06%)
9	20 (20.4%)			
10	17 (17.34%)			2 (2.04%)
11	11 (11.22%)			
12	10 (10.2%)			
13	3 (3.06%)			
14	2 (2.04%)	1 (1.02%)		
15	2 (2.04%)	3 (3.06%)		
16	2 (2.04%)	3 (3.06%)		
17		2 (2.04%)		
18		2 (2.04%)		
19		4 (4.08%)		
20		1 (1.02%)		1 (1.02%)
21		4 (4.08%)		
22		7 (7.14%)		
23		3 (3.06%)	3 (3.06%)	
24		8 (8.16%)	4 (4.08%)	
25		11 (11.22%)	2 (2.04%)	
26		9 (9.18%)	3 (3.06%)	
27		6 (6.12%)		
28		14 (14.28%)	4 (4.08%)	
29		11 (11.22%)		
30	1 (1.02%)	7 (7.14%)	6 (6.12%)	
31		1 (1.02%)	5 (5.1%)	
32		1 (1.02%)	5 (5.1%)	
33			10 (10.2%)	
34			11 (11.22%)	
35			6 (6.12%)	
36			6 (6.12%)	
37				
38			5 (5.1%)	
39			8 (8.16%)	
40			5 (5.1%)	
41			3 (3.06%)	
42			4 (4.08%)	
43			2 (2.04%)	
44			3 (3.06%)	
45				
46			2 (2.04%)	
47				
48				
49			1 (1.02%)	
50				
Median value ± standard deviation/years	9.38 ± 3.08	24.79 ± 4.18	34.51 ± 5.81	2.71 ± 2.713

In regard to the behavioral factors that potentially favor the development of OAB, 23 patients (23.46%) were smokers (95% CI: 0.1549644–0.3310564), with a 14.54 pack-years index, and 13 patients (13.26%) had excessive alcohol consumption (95% CI: 0.07256431–0.21615400), as shown in Table 2. As for the professional factors that potentially favor the development of OAB (Table 2), 25 patients (25.51%) had psychological factors (anxiety, social stress, or professional stress) (95% CI: 0.1723863–0.3531425) and 7 patients (7.14%) had special working environments (cold or humidity). In regard to associated pathologies affecting patients with OAB (Table 2), 22 patients (22.44%) had a history of lower urinary tract infection (95% CI: 0.1463523–0.3199184), 7 patients (7.14%) had a history of duodenal ulcers, 5 patients (12.19%) had a history of cesarean operation, 5 patients (5.10%) had a history of hypertension, 4 patients (4.08%) had a history of diabetes mellitus, and one patient (1.02%) had a history of multiple cystic mastitis.

**Table 2:** Risk factors

Type of risk factor	Risk factor	Patient number	Patient percentage
Behavioral	Smoking	23	23.46
	Excessive alcohol consumption	13	13.26
Professional	Psychological	25	25.51
	Special working environments	7	7.14
Associated pathologies	Lower urinary tract infection	22	22.44
	Duodenal ulcers	7	7.14
	Cesarean operation	5	5.1
	Hypertension	5	5.1
	Diabetes mellitus	4	4.08

Because of the short period of the study, a small number of cases were included. Our intention was to perform a limited observational study to ascertain whether there is statistical support for future larger studies on this topic. 

## 4. Discussion 

Goessaert et al. highlighted the fact that mictional urgency, daytime frequency, urinary incontinence, and nocturia occurred in adults who had exhibited NE in childhood at notable percentages: 17%, 8%, 25%, and 35%, respectively. In our study the incidence of patients suffering from OAB who had previously displayed NE symptoms seems to be considerable, 34.38% of all cases; this percentage cannot be ignored or considered a coincidence. Thus, it may be considered that the remission of NE is not equivalent to the resolution of the observed mictional dysfunctions, but rather, for a number of patients, it is only the initial stage of the development of new bladder ailments that will manifest in young adulthood [17].

There was a slight majority of male patients suffering from OAB in our study group (58.16%), which contradicts previous reports of a minimal female predominance [8,9] but corresponds to previous reports in the literature regarding NE male predominance. Could this constitute an argument in favor of a central type of neurogenic etiopathogeny of overactive bladder? 

The fact that the greater majority of patients suffering from NE were from an urban area (75.51%) suggests either the existence of acquired urban factors (social, professional, or nutritional) that could cause the recurrence of mictional dysfunctions, or lower ease of access to clinical specialists in rural areas, mainly due to educational, social, or cultural factors [22]. Because the majority of statistically significant research is performed in economically advanced countries [19,20], which have a predominantly urban population, specific information regarding the environmental origins of OAB patients is lacking. In the case of predominance of risk factors (behavioral and psychological) specific to urban lifestyles, a predominance of urban patients (15.4%) makes sense in comparison to 13.3% of patients with rural origins [23]; however, reports should not be so unbalanced. In our country, which has a small urban majority (55.2%), this considerably high percentage of patients from urban origins in this study (75.51%) could be a serious argument for a series of behavioral and psychological factors that are much more frequently associated with urban life to be triggering factors of new urinary dysfunctions. 

The median patient age at the moment of NE remission was 9.83 years, with a range of 6 to 30 years. The highest number of cases, 70 (71.42%), showed NE remission at ages of 8, 9, 10, 11, and 12. In most cases, NE remitted at the age of 12, and these values correspond to those given in the current literature, with one exception: the patient whose NE remission occurred at the age of 30 [24].

The time span between NE remission and the diagnosis of OAB ranged from 14 to 32 years, with a median value of 24.79 years, but because of the absence, as far as we know, of relevant data on this topic in the literature, these results cannot be compared or analyzed. We maintain our position that there could be one or possibly several triggering factors acting at random time intervals to cause bladder dysfunctions, but confirming this assumption will require additional clinical, preferably prospective, studies performed on sufficiently large patient study groups to obtain statistically significant results.

The average age of patients at the moment of OAB diagnosis was 34.51; however, taking into account the median time interval between the initiation of symptomology and diagnosis, 2.71 years, the median age at which OAB began affecting the patients was 31.80 years, a values included in the 25–35 years interval. In another study, maximum OAB incidence was observed in patients between 35 and 44 years of age and between 45 and 54 years, with values of 22.8% and 17.2%, respectively [25]. Thus, we may wonder whether the patients who displayed NE symptoms in childhood developed OAB earlier than those who did not, as this would indicate that NE is a predisposing factor of OAB.

Identifying the risk factors behind OAB development is a commonly undertaken goal for researchers and clinicians. However, the opinions of the specialists in this field on OAB’s etiopathogeny differ and sometimes are even divergent. Traditionally, it was believed that age, sex, low levels of education, obesity, physical labor, alcohol consumption, multiple parities, and vaginal delivery were risk factors of OAB [26]. A contradicting study claimed that obesity, alcohol consumption, physical labor, and smoking are not risk factors of OAB in men and that restrictions on alcohol (beer) and large consumption of potatoes could be risk factors of OAB [27]. The same authors claimed that the risk factors of OAB in women are obesity, smoking, and consumption of effervescent beverages [28]. The analysis of our study group reveals several common features with those in the current literature, namely the presence of smokers (23.46%), alcohol consumers (13.26%), prior lower urinary tract infections (22.44%), and patients who underwent obstetric interventions (12.19%). We especially wish to point out the psychological factors that have been described in the specialty literature as determinant or associated elements for OAB [29,30], including anxiety or socioprofessional stress, which affected 25.51% of patients in our study. We would also like to draw special attention to the seven cases (7.14%) of patients who complained of special work environmental conditions (cold or humidity) and who claimed that their vesical pains began after a period of time spent in these conditions.

OAB has great impact on the health-related quality of life of patients and, as such, exerts a significant financial burden. In a study performed in six economically advanced countries, Irwin estimated that the direct annual costs of OAB are 3.9 billion USD, that home treatment costs reach up to 4.7 billion USD, and absence from the work place translates into an additional 1.1 billion USD [31]. The identification of a causal relationship between NE and OAB would not only have great impact on multiple medical fields (general practitioners, pediatricians, neurologists, urologists, etc.), but would also allow the parents of children suffering from NE to be informed about the possible bladder dysfunctions that their children may face in adulthood. This would aid in early diagnosis and treatment of OAB and thus a reduction of costs associated with OAB.

Finally, a clear limitation of this study is the small number of included cases. However, this study generated accurate data because a standard protocol involving clinical, laboratory, and imagistic investigations for the diagnosis of OAB was followed and all the cases in the study group were included only by the authors, without using a cross-sectional telephone, postal, or Internet survey. 

In conclusion**, **the statistical analysis revealed that 34.38% of the patients diagnosed with OAB had previously suffered from NE, which suggests a relationship of the physiopathological causation of these two conditions. A great majority of patients (75.51%) came from an urban environment, which suggests the possible existence of acquired urban triggering factors of OAB (nutritious, social, or professional). In this cohort, complaints about the bladder issue began at the age of 32, which is earlier than reported in the literature. Among the risk factors of OAB we would like to underline the psychological factors (anxieties and socioprofessional stress) and environmental factors (low temperatures and humidity in the work place) that were observed in over a third of all cases. Considering the major impact that OAB has on health-related quality of life and on the budgets of health institutions, we believe the possible causal relationship between NE and OAB warrants additional studies.
